# Supplementary material for: ATR is a multifunctional regulator of male mouse meiosis
Source: Nat Commun. 2018 Jul 5;9:2621. doi: 10.1038/s41467-018-04850-0 (PMC6033951; doi:10.1038/s41467-018-04850-0)
Supplement: Supplementary file 1 — Supplementary Information [file 41467_2018_4850_MOESM1_ESM.pdf]

Supplementary Information

ATR is a multifunctional regulator of male mouse meiosis

Widger *et al*.

Supplementary Figure 1

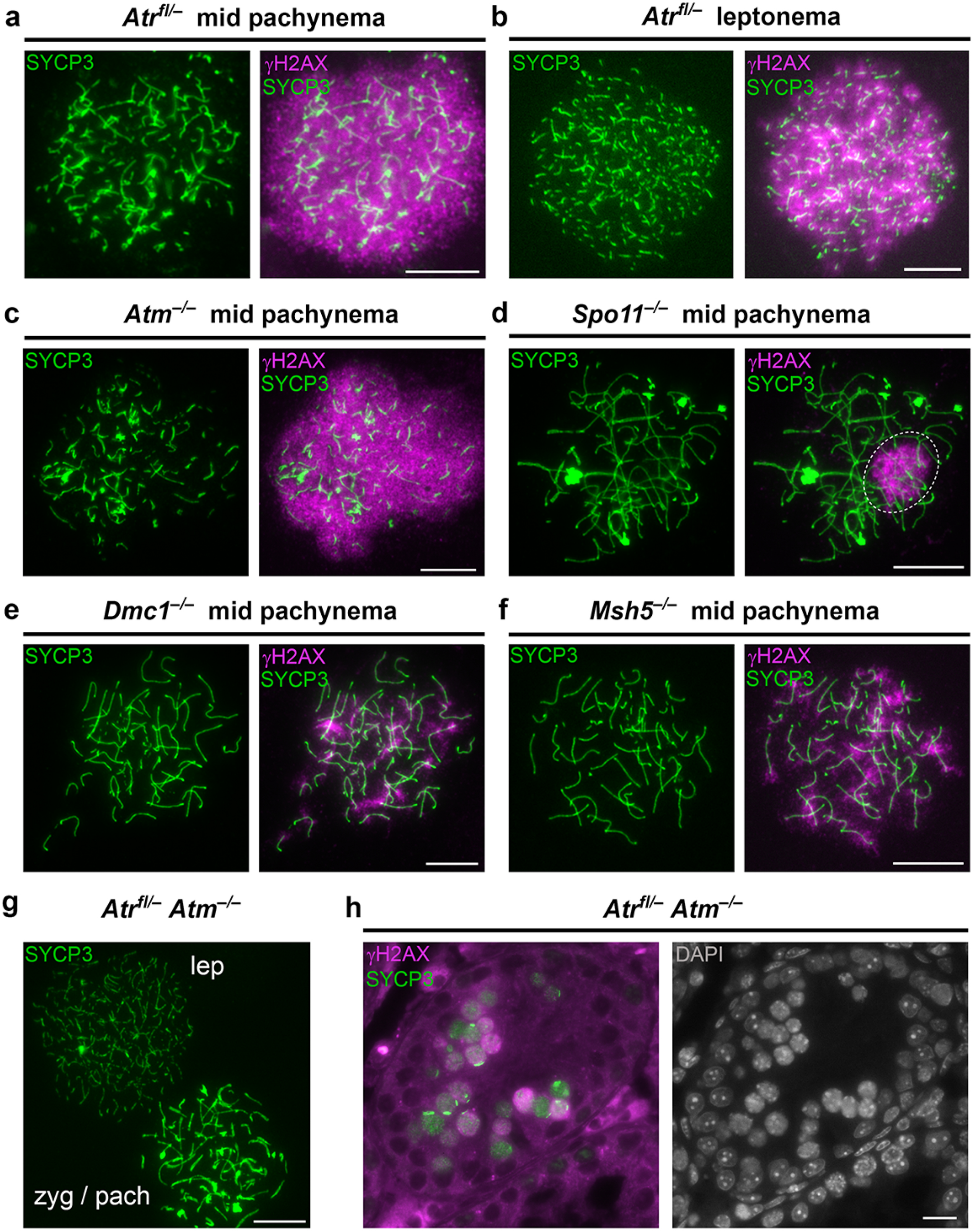

Supplementary Figure 1: Mid-pachytene axial morphology and γH2AX staining in meiotic mutants. Immunostaining for γH2AX (magenta) and SYCP3 (green) in (**a**) mid pachytene Atrfl/– cells (n = 3 males, 60 cells), (**b**) leptotene Atrfl/– cells (n = 1 male, 50 cells), (**c**) mid pachytene Atm–/– cells (n = 2 males, 50 cells), (**d**) mid pachytene Spo11–/– cells (n = 2 males, 40 cells; pseudosex body circled), (**e**) mid pachytene Dmc1–/– cells (n = 2 males, 40 cells) and (**f**) mid pachytene Msh5–/– cells (n = 2 males, 40 cells). (**g**) in Atrfl/– Atm–/– males, leptotene cells can be identified (upper cell). Of the remaining cells, 14% show the morphology typical of zygonema (see Fig. 2c), while the others cannot be unambiguously identified as in zygonema or pachynema due to axial fragmentation (n = 2 males, 80 cells). (h) Atrfl/– Atm–/– testis section showing that pachytene spermatocytes with nucleus-wide γH2AX staining. Scale bar in (h) 20 μm, other scale bars 10 μm.

Supplementary Figure 2

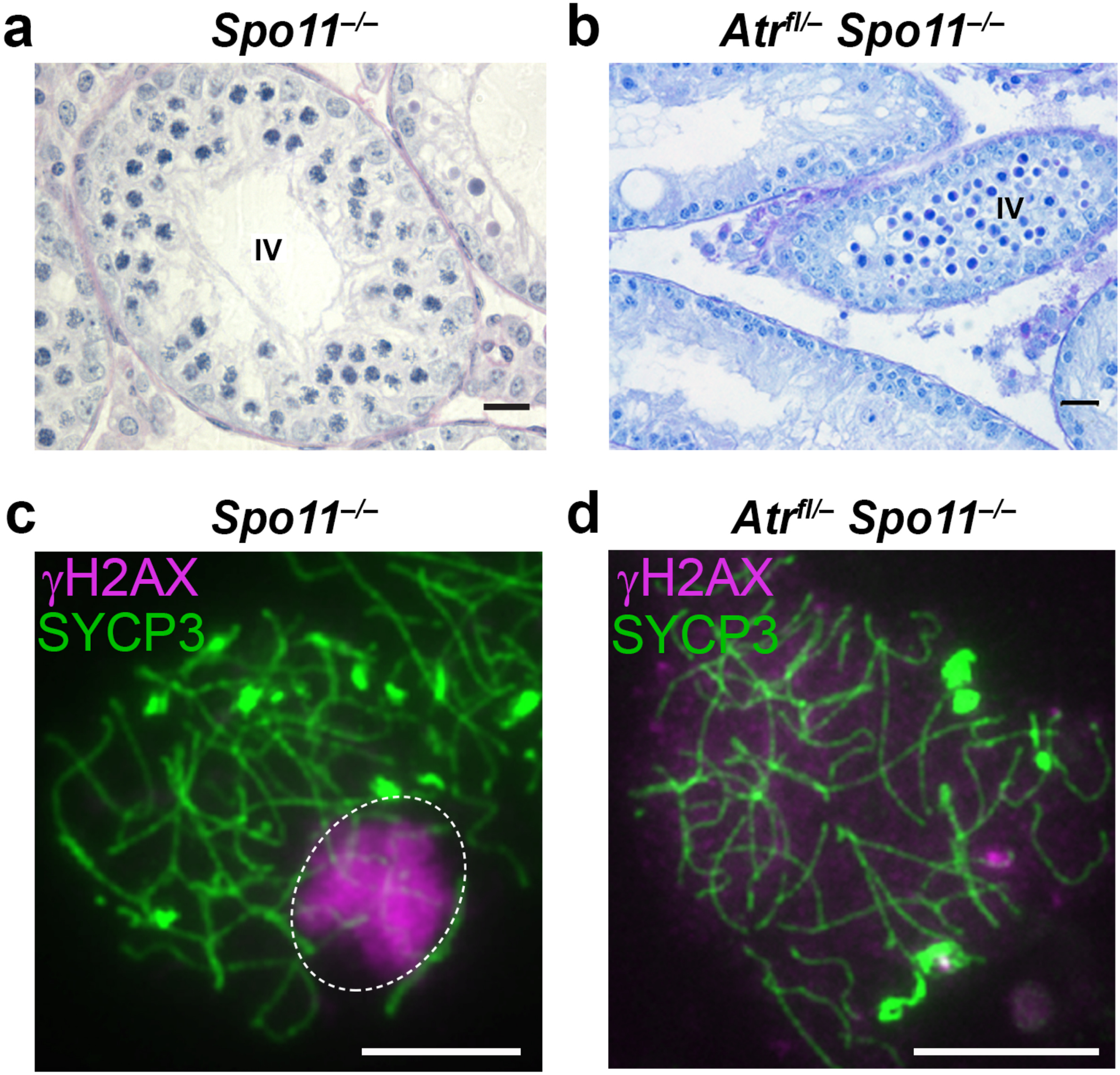

Supplementary Figure 2: Atr deletion in Spo11–/– mice ablates pseudosex body formation but not stage IV germ cell elimination. Periodic Acid-Schiff stained stage IV testis sections from (**a**) Spo11–/– and (b) Atrfl/– Spo11–/– males. The Atrfl/– Spo11–/– stage IV section contains more arrested pachytene cells is because it is sectioned tangentially. As a result, there are also two layers of spermatogonia and Sertoli cell nuclei. The fact that the more advanced tubules above (containing preleptotene cells) and below (containing B spermatogonia) contain no spermatocytes demonstrates that all germ cells in Atrfl/– Spo11–/– males are eliminated at stage IV. The pseudosex body is present in 94% of Spo11–/– mid pachytene cells (n = 2 males, 50 cells; **c**) but in no Atrfl/– Spo11–/– mid pachytene cells (n = one male, 50 cells; **d**). Scale bar 20 μm in (**a**,**b**) and 10 μm in (**c**,**d**).

Supplementary Figure 3

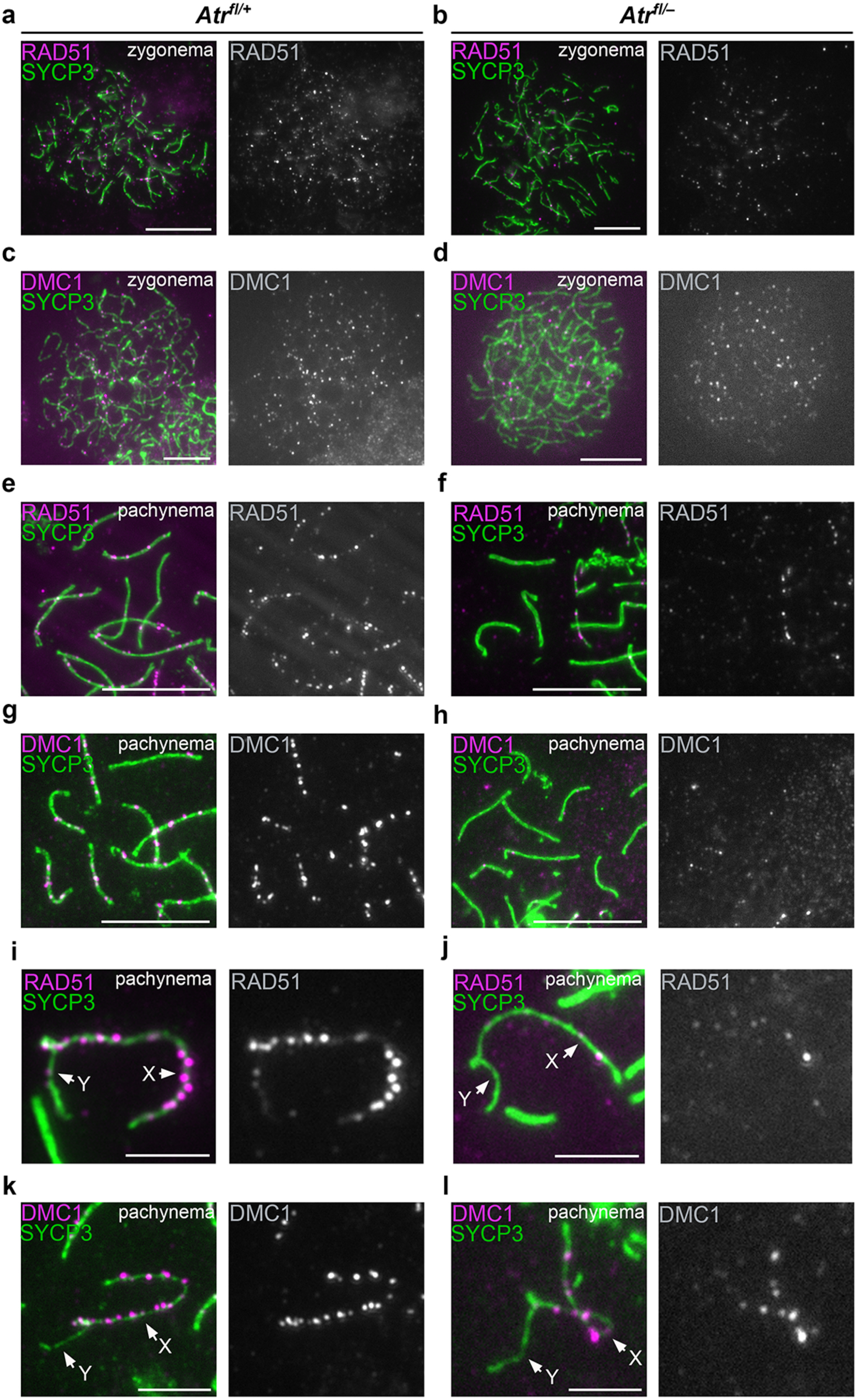

Supplementary Figure 3: ATR regulates DSB marker counts during zygonema and pachynema. Analysis of focus counts using SYCP3 (green) and early recombination markers (magenta) in Atrfl/+ and Atrfl/– males: (**a**,**b**) RAD51 at mid zygonema, (**c**,**d**) DMC1 at mid zygonema, (e,f) RAD51 on autosomes at early pachynema, (**g**,**h**) DMC1 on autosomes at early pachynema, (**i**,**j**) RAD51 on the X chromosome at early pachynema, (**k**,**l**) DMC1 on the X chromosome at early pachynema. Scale bar 10 µm in (a-h), 5 µm in (**i**-**l**).

Supplementary Figure 4

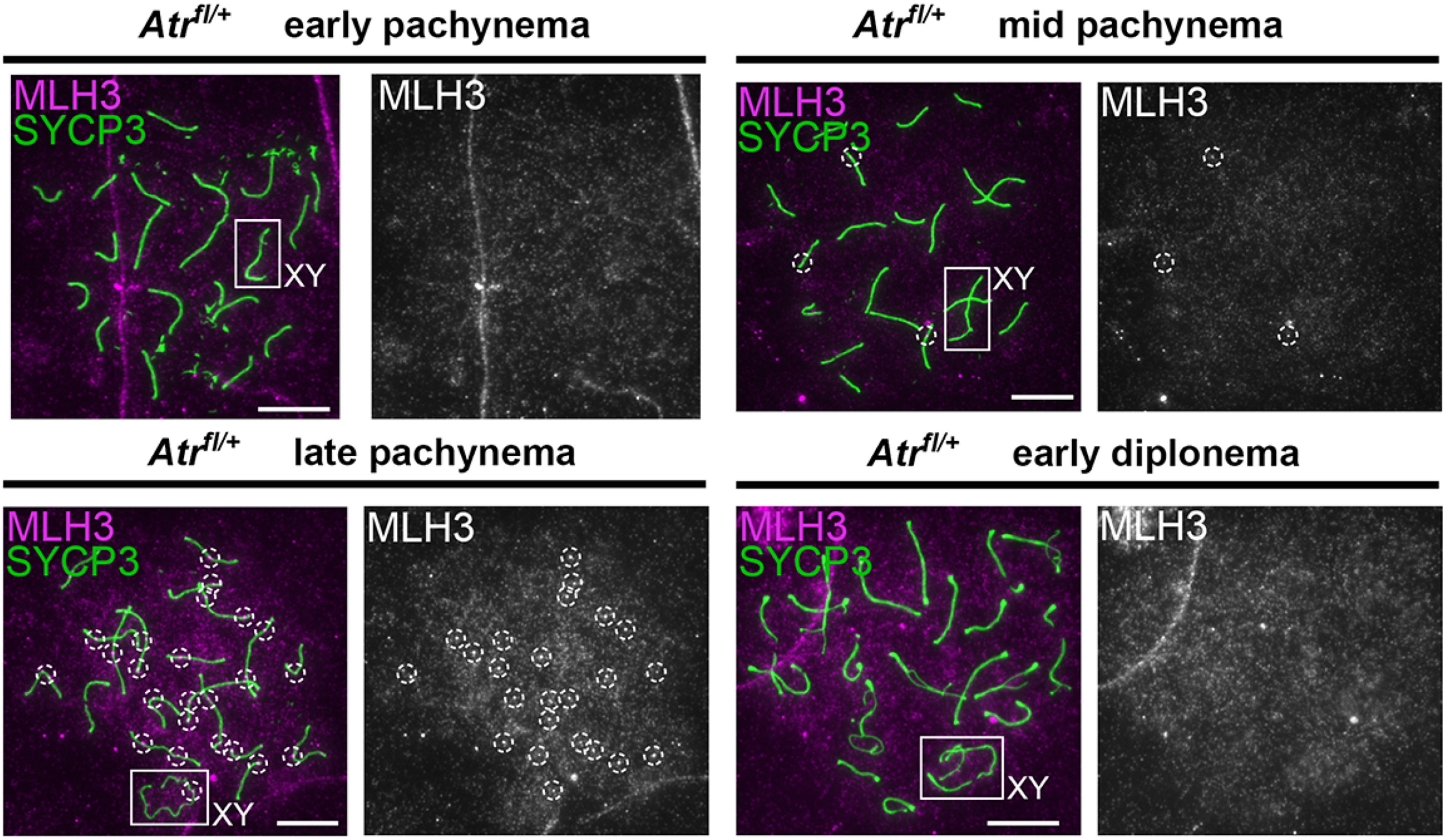

Supplementary Figure 4: Appearance of the late recombination marker MLH3 during pachynema in controls. Staining for MLH3 (magenta) and SYCP3 (green). MLH3 cannot be detected on the autosomes or PAR of the XY bivalent (boxed) at early pachynema, and few foci (circled) are apparent at mid pachynema. At late pachynema foci are present both on the autosomes and the PAR of the XY bivalent. At early diplonema, MLH3 foci have disappeared (n = 1 male, 10 cells at each stage) Scale bar 10 µm.

Supplementary Figure 5

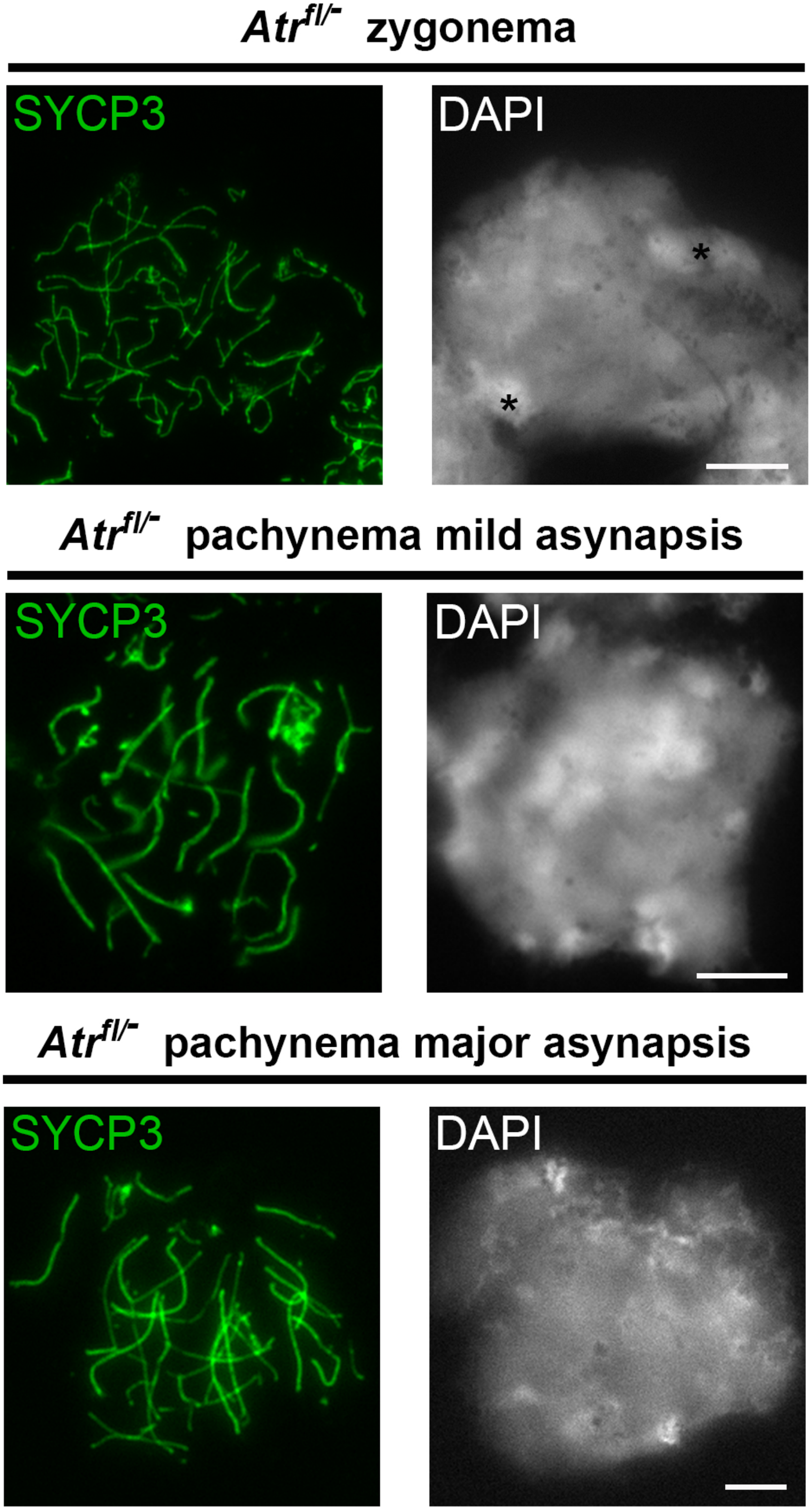

Supplementary Figure 5: Substaging of spermatocytes in Atrfl/– males. SYCP3 (green) and DAPI (grey). Zygotene spermatocytes contain long thin, discontinuous axial elements, few regions of centromeric heterochromatin (black asterisks) and more homogeneous DNA staining. Pachytene spermatocytes contain shorter, fully formed axial elements, multiple smaller regions of centromeric heterochromatin and more heterogeneous DNA staining. Shown are two pachytene spermatocytes, one with mild asynapsis and another with major asynapsis. The spermatocyte with major asynapsis can be readily distinguished from the zygotene spermatocyte based on the above criteria, and because in the pachytene spermatocyte there is asynchrony in synapsis, with some homologues fully synapsed and others, largely asynapsed. Scale bar 10 µm.

Supplementary Figure 6

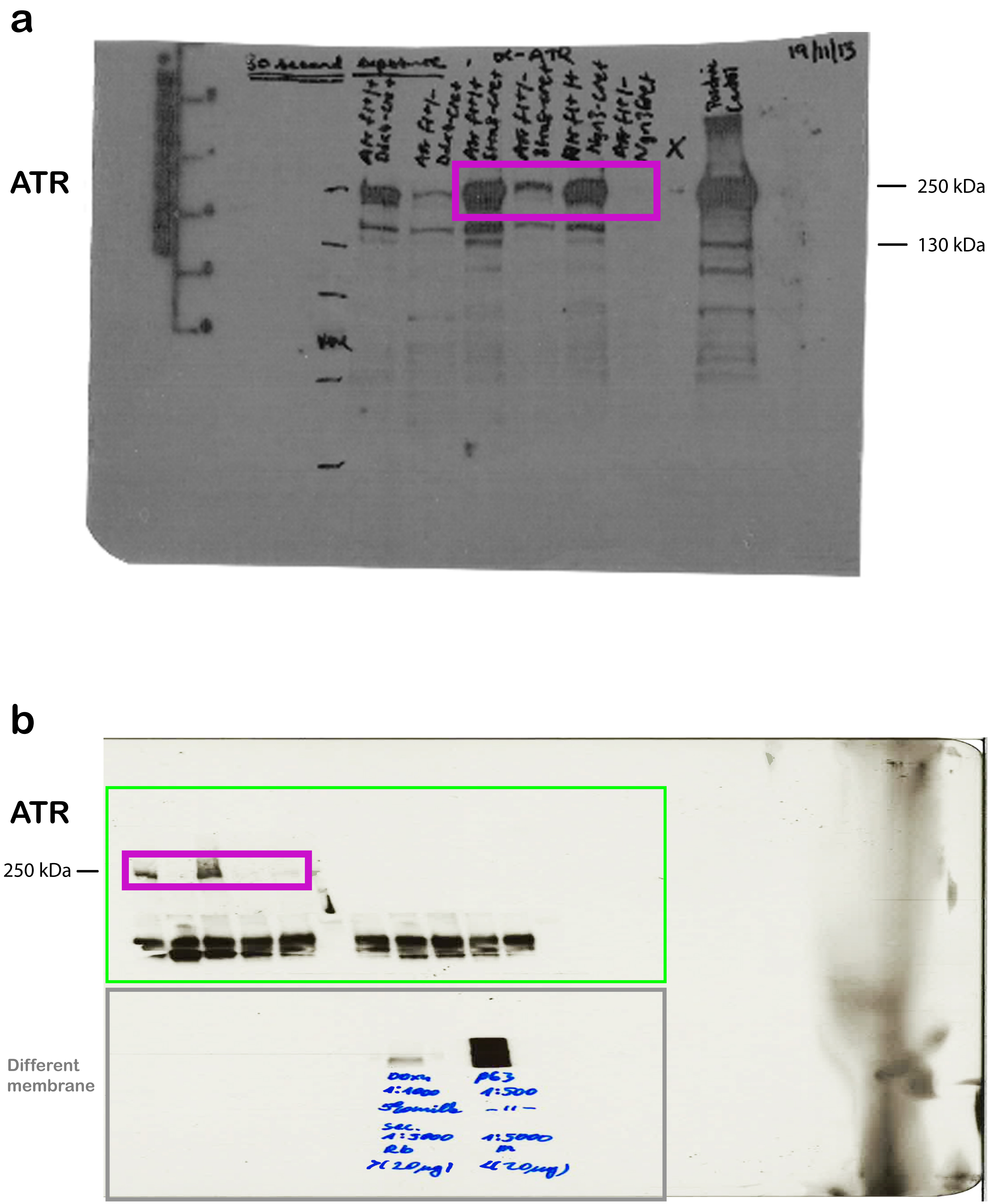

Supplementary Figure 6 (continued)

c

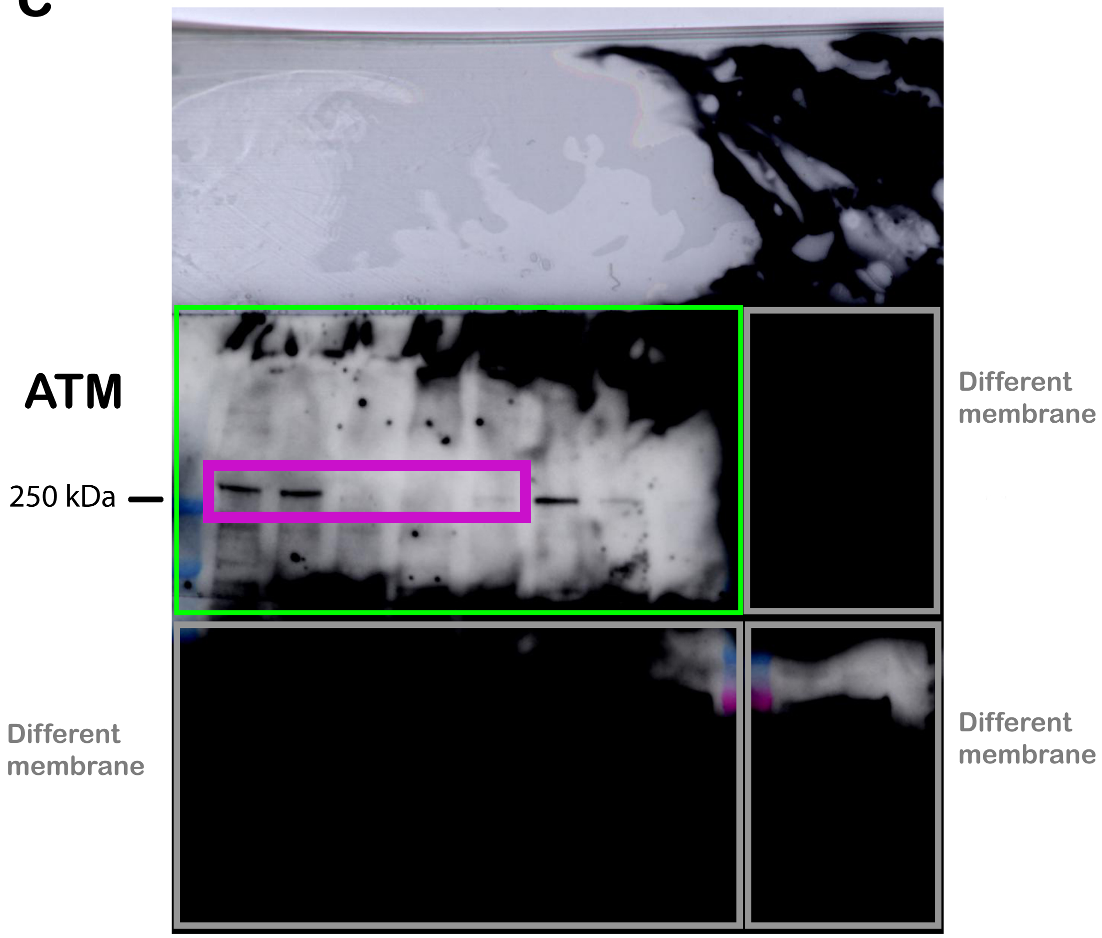

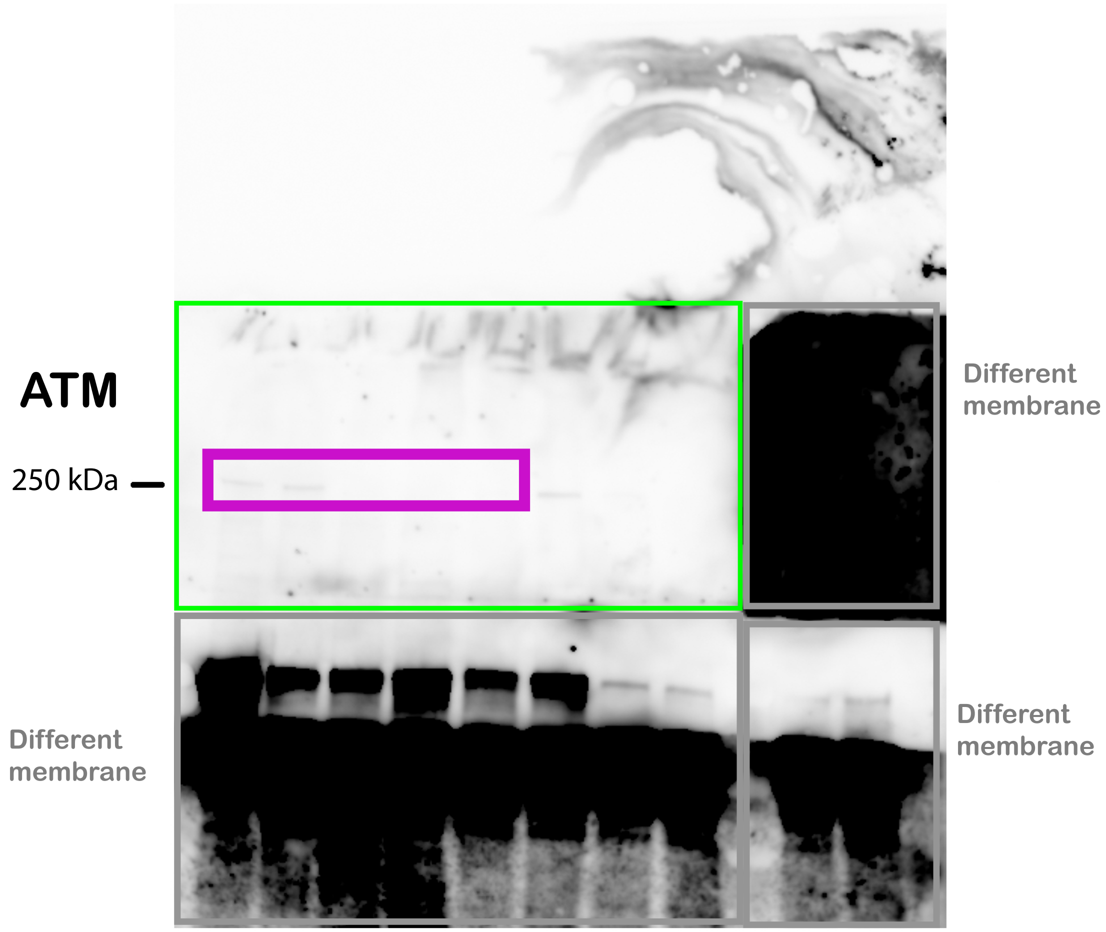

Supplementary Figure 6. Uncropped images of blots with molecular weight markers. Reference to figures is provided for each presented blot : (**a**) related to Figure 1a, (**b**) related to Figure 2a, (**c**) related to Figure 2b. Purple rectangle shows the area presented in related figure; green rectangle identifies the area of relevant membrane. Grey rectangles represent different membranes not relevant to this study. (**c**) shows longer exposure blot at the top and shorter exposure blot at the bottom. Note in panel (**a**) that only high molecular weight bands are rectangled in purple (predicted molecular weight of ATR = 317 kDa). Lower molecular weight bands are also reduced in the ATR conditional knockouts and may therefore represent other ATR isoforms.
